# Mammary Abscess

**Published:** 1875-09

**Authors:** 


Mammary Abscess.—The following is a very simple plan of treatment, and it is claimed that it is much more satisfactory than strapping the breast with adhesive plaster, counter openings, injecting the sinuses with iodine, etc., all of which measures have been extensively resorted to, especially in the country.

What the surgeon desires to do in these cases is to evacuate the abscess thoroughly, at the same time to press the walls of the abscess together, and then retain them in contact. These are substantially the indications to be met, so far as local measures are concerned; for if the two surfaces can be held thoroughly in contact during forty-eight hours, granulations will spring up which will unite them, and the abscess will be closed.

The plan of treatment recommended meets all these indications and accomplishes the desired results without giving rise to pain, but on the contrary, those who had the dressing applied said that it was rather pleasant. It is a plan of dressing within the reach of any practitioner, and can be applied to any breast, no matter how tender. It affords gentle but firm pressure, and holds the walls of the abscess in contact until union takes place.

Take a piece of coarse sponge, large enough to cover the breast, and about four inches thick; make it a trifle concave or cup-shaped upon one surface, thoroughly cleanse it in boiling water, being very careful to remove all particles of sand, and, while wet, place it between two pieces of board and subject it to at least fifty pounds pressure. At the end of twenty hours it will be dry and ready for use.

First cover the breast with a moderately thick layer of oakum, which readily absorbs the pus, and at the same time has an antiseptic influence.

Coarse lint may be used, but it is not nearly as pervious to pus as is the oakum, and must be watched more closely, lest free discharge from the abscess is prevented by it. It is important to get the breast up and support it, and this is done by raising it with the hand, before applying the sponge. It is then retained in position by applying the bandage, which is to hold the sponge in place, sufficiently tight, and in the proper direction to accomplish this purpose.

Always leave the upper edge of the sponge uncovered, so that it can be easily wet, for it is almost impossible to wet the sponge through several thicknesses of a tightly-applied muslin roller. The dressing should not be removed for two or three days, and then another sponge should be immediately applied, and thus the dressing kept up for at least eight or ten days, in order to be certain that the surfaces of the abscess are firmly united. The question arises whether this dressing should be applied when there is considerable milk in the breast. There need be no hesitation upon this point, for the milk is readily taken care of by the absorbents, and does no harm. A single sponge may be wet two or three times before its elasticity will be expended, and then it is necessary to make the second application.—*Med. Record.*

